# Identification of ATP-Binding Regions in the RyR1 Ca^2+^ Release Channel

**DOI:** 10.1371/journal.pone.0048725

**Published:** 2012-11-07

**Authors:** Olga B. Popova, Mariah R. Baker, Tina P. Tran, Tri Le, Irina I. Serysheva

**Affiliations:** Department of Biochemistry and Molecular Biology, The University of Texas at Houston Medical School, Houston, Texas, United States of America; Cinvestav-IPN, Mexico

## Abstract

ATP is an important modulator of gating in type 1 ryanodine receptor (RyR1), also known as a Ca^2+^ release channel in skeletal muscle cells. The activating effect of ATP on this channel is achieved by directly binding to one or more sites on the RyR1 protein. However, the number and location of these sites have yet to be determined. To identify the ATP-binding regions within RyR1 we used 2N_3_ATP-2′,3′-Biotin-LC-Hydrazone (BioATP-HDZ), a photo-reactive ATP analog to covalently label the channel. We found that BioATP-HDZ binds RyR1 specifically with an IC_50_ = 0.6±0.2 mM, comparable with the reported EC50 for activation of RyR1 with ATP. Controlled proteolysis of labeled RyR1 followed by sequence analysis revealed three fragments with apparent molecular masses of 95, 45 and 70 kDa that were crosslinked by BioATP-HDZ and identified as RyR1 sequences. Our analysis identified four glycine-rich consensus motifs that can potentially constitute ATP-binding sites and are located within the *N*-terminal 95-kDa fragment. These putative nucleotide-binding sequences include amino acids 699–704, 701–706, 1081–1084 and 1195–1200, which are conserved among the three RyR isoforms. Located next to the *N*-terminal disease hotspot region in RyR1, these sequences may communicate the effects of ATP-binding to channel function by tuning conformational motions within the neighboring cytoplasmic regulatory domains. Two other labeled fragments lack ATP-binding consensus motifs and may form non-canonical ATP-binding sites. Based on domain topology in the 3D structure of RyR1 it is also conceivable that the identified ATP-binding regions, despite their wide separation in the primary sequence, may actually constitute the same non-contiguous ATP-binding pocket within the channel tetramer.

## Introduction

Type 1 ryanodine receptor (RyR1) is an intracellular ligand-gated Ca^2+^ release channel located in the sarcoplasmic reticulum (SR) of skeletal muscle cells. RyR1 mediates the release of Ca^2+^ from the SR into the cytoplasm and thereby plays a fundamental role in the generation of Ca^2+^ signals in excitation-contraction coupling resulting in muscle contraction. RyR1 forms a macromolecular complex comprised of four subunits of ∼560 kDa each, in combination with an array of modulatory cellular proteins [1], [2], [3]. Each RyR1 subunit consists of a cytoplasmic *N*-terminal region (∼80% of the protein mass), at least six membrane-spanning segments and a small cytoplasmic *C*-terminal region [4], [5], [6]. RyR1 function is intricately regulated by multiple intracellular ligands (e.g. Ca^2+^, ATP, caffeine, Mg^2+^, calmodulin, FKBP12) that interact with the channel complex in a dynamic manner to provide functional feedback [7], [8], [9], [10]. Most of these ligands/modulators are known to bind within the large cytoplasmic region of RyR1 and allosterically regulate the opening/closing of the channel gate.

Adenine nucleotides, including ATP, are among the major modulators of the Ca^2+^ release channel function. Similar to several other ion channels, ATP acts as a non-hydrolyzed ligand involved in the activation of RyR1 with an EC_50_ in the low millimolar range when in the presence of micromolar Ca^2+^ [11], [12], [13], [14]. ATP enhances Ca^2+^ release from the SR and stimulates Ca^2+^ release through RyR1 channels incorporated into lipid bilayers [15], [16], [17], [18], [19], [20], [21]. The three RyR isoforms, each with different tissue expression profiles, exhibit distinct modulation by ATP and related adenine nucleotides [22], [23], which, in part, underlies the varied characteristics of Ca^2+^ release mechanisms found in different muscle types.

At present, the molecular mechanism of ATP-binding and ion channel activation in RyRs is poorly understood. Several studies indicate that the activating effect of ATP occurs through the direct binding to one or more sites on RyR1 that function cooperatively in the tetrameric channel [15], [16], [24]. The number of accessible ATP-binding sites in RyR1 has been shown by ESR spectroscopy to directly correlate with the functional state of the channel induced by channel-specific ligands. Two ATP-binding sites per RyR1 monomer were identified in the presence of micromolar Ca^2+^ and eight ATP-binding sites were identified in the presence of caffeine or ryanodine [25], [26]. The non-hydrolyzable analog of ATP, AMP-PCP, was found to increase the open probability of RyR1 channels incorporated into planar lipid bilayers confirming that the activating effect of ATP was not the result of phosphorylation of the channel or closely associated proteins [19], [27].

Furthermore, long-range conformational changes in the 3D structure of RyR1 in the presence of 100 µM Ca^2+^ and 1 mM AMP-PCP have been revealed by low-resolution electron cryo-microscopy (cryo-EM) studies [28]. Thus, it was postulated that the binding of ATP to RyR1 induces conformational changes stabilizing the open channel state [29]. However, the structural basis for ATP modulation of RyR1 remains poorly understood due to a lack of knowledge about the location and composition of the ATP-binding sites, their spatial relation with respect to the ion-conducting channel pore and the nature of the conformational changes that ensue after ATP-binding.

The RyR1 protein sequence is predicted to contain several regions with the Walker-A and a partial Walker-B nucleotide-binding consensus motifs, GXGXXG and [R/K]XXXGXXXL, respectively, originally identified in a diverse set of ATP-binding proteins that involved the catalysis of ATP [30], [31]. In addition, several conserved sequences in RyR1 conform to the ATP-binding consensus motifs for the ATP-binding site found in the chaperonin GroES, Y(G/A/S/T)(V/G)(K/T/Q/S/N), which does not hydrolyze ATP [32]. Therefore, it has been generally proposed that one or several of the predicted ATP-binding motifs in RyR1 may be responsible for ATP-dependent channel activation [4], [5], [9], [33], [34]. Nevertheless, no experimental evidence exists demonstrating the direct involvement of these sequences in ATP binding.

In the present study, we utilized a biotinylated azido analog of ATP to photoaffinity label and localize the ATP-binding sites in the primary sequence of RyR1. Three regions were identified following limited proteolysis and sequence analysis of the labeled proteolytic fragments. Given their distant locations in the primary structure of RyR1, these regions might constitute the same ATP-binding pocket in the 3D structure of RyR1. Furthermore, four ATP-binding consensus sequences were identified within the labeled *N*-terminal fragment that can constitute the putative ATP-binding site. The identified ATP-binding region is located in close proximity to one of the disease hotspot regions in RyR1. This finding provides an essential window into understanding how ATP-binding can affect receptor function in normal and diseased conditions.

## Materials and Methods

### Materials

3-[(3-Cholamidopropyl) dimethylammonio]-1-propanesulfonate (CHAPS) was obtained from Anatrace (Manmee, OH). Na_2_-ATP, bovine pancreas trypsin, soybean trypsin inhibitor and protease inhibitors (leupeptin, pepstatin A, phenylmethyl-sulfonyl fluoride, aminobenzamidine, aprotinin) were obtained from Sigma-Aldrich (St. Louis, MO). 2-azidoadenosine 5′-trisphosphate 2′,3′-biotin-long-chain-hydrazone (BioATP-HDZ) was from Affinity Photoprobes (Lexington, KY). IRDye800CW-streptavidin was from LI-COR Biosciences (Lincoln, NE).

### Antibodies

A polyclonal anti-RyR1 antibody against the amino acid sequence 416–434 was produced by Cocalico Biologicals, Inc., (Reamstown, PA) and affinity-purified using protein A/G sepharose, GE Healthcare (Pittsburgh, PA). Monoclonal anti-calsequestrin 1 (4i392) and anti-SERCA2 (IID8) antibodies were obtained from Santa Cruz Biotechnology, Inc., (Santa Cruz, CA). Anti-RyR1 monoclonal antibody MA3-925 was obtained from ThermoScientific (Rockford, IL). Secondary antibodies, IRDye680RD anti-mouse IgG and IRDye800CW anti-rabbit IgG were obtained from LI-COR Biosciences (Lincoln, NE).

### SR Membrane Preparation

Microsomal membranes were prepared from rabbit skeletal muscle as described earlier [35]. To obtain the SR membranes enriched with RyR1, the microsomal membranes were separated on a sucrose step gradient of 20, 25, 30, 35 and 40% sucrose. The SR membranes from the 35/40% interface contained most of RyR1 and were used for RyR1 purification and photoaffinity labeling experiments.

### Purification of RyR1

RyR1 was purified from the SR membranes as described earlier [36]. Briefly, the SR membranes were solubilized with 2% CHAPS in 50 mM MOPS (pH 7.4), 185 mM NaCl, 1 mM DTT, 0.1 M EGTA. The solubilized channel protein was then purified by a two-step procedure: ion-exchange chromatography on a DEAE-Trisacryl M column followed by centrifugation through a 5–20% linear sucrose density gradient. Protease inhibitors were used throughout the protein isolation.

### Photo-Reactive Labeling

SR membranes were diluted to 1 or 10 µg/µl in 50 mM MOPS (pH 7.4), 300 mM NaCl, 1 mM EGTA and 10 µM BioATP-HDZ was added. Purified RyR1 was used at ∼2 µg/µl. The samples were incubated for 1 h at 4°C in UV-protected tubes and then irradiated at 254 nm for 90 sec at room temperature in a UV crosslinker (Stratalinker, Model 2400) at an intensity of 762.3 mW/cm^2^. For competition assays protein samples were pre-incubated with 0.01–10 mM ATP (in SR membranes) or with 0.01–50 mM ATP (in purified RyR1) for 1 hour at 4°C before the addition of 10 µM BioATP-HDZ. BioATP-HDZ labeling was identified using IRDye800CW-streptavidin overlay, for which the fluorescence signal was measured at 800 nm and normalized to the corresponding signal from Coomassie Brillaint Blue (CBB) stained gels, measured at 700 nm. The competition assay was analyzed using the equation *y = (F–F_0_)/F_max_*, where *F* is the IRDye800CW-streptavidin fluorescence signal, *F_0_* is a background signal and *F_max_* is the signal with no ATP added. Total protein concentration was estimated by BioRad protein assay with the absorbance measured at 595 nm.

### Trypsin Digestion

Limited proteolysis and isolation of the RyR1 macromolecular complex was performed as described previously [37]. Briefly, SR membranes (10 µg/µl) were digested with trypsin for 5 min at 37°C at a trypsin to protein ratio of 1∶1000. The reaction was terminated by the addition of 10-fold excess soybean trypsin inhibitor. SR membranes were then solubilized in 2% CHAPS for 30 min on ice. The RyR1 proteolytic complexes were semi-purified by centrifugation over a 5–20% linear sucrose gradient at 24,000 rpm for 17 h at 4°C in a SW28 rotor (Beckman). 1 ml fractions of sucrose gradients were collected from the bottom of the tube and analyzed using Bio-Rad protein assay, SDS-PAGE and Western blot.

Purified RyR1 (2 µg/µl) in 50 mM MOPS (pH 7.4), 0.4% CHAPS, 300 mM KCl, 1 mM EGTA was digested with trypsin for 10 min at 37°C at a trypsin to protein ratio of 1∶100. The reaction was terminated by 10-fold excess of soybean trypsin inhibitor. Digested fragments were analyzed by SDS-PAGE and Western blot.

### Near-Infrared Imaging

SDS-PAGE was performed according standard Laemmli procedure [38]. Visualization of the photoaffinity labeled polypeptides was performed by IRDye800CW-streptavidin overlay either in-gel or after an over-night transfer to Immobilon-FL (Millipore). Gels were fixed for 10 min in a solution containing 50% methanol and 10% acidic acid, washed for 15 min in water, incubated with 1 µg/ml IRDye800CW-streptavidin in PBS-T (phosphate buffered saline with 0.01% Tween-20) for 15 min and followed by a wash in PBS for 10 min before scanned at 800 nm. Gels were then stained with CBB and scanned at 700 nm. Immobilon-FL membranes were fixed with 100% methanol and washed with PBS-T for 15 min followed by incubation with 1 µg/ml IRDye800CW-streptavidin in PBS-T for 15 min. The membranes were washed again with PBS-T for 10 min and PBS for 5 min before they were scanned at 800 nm. Precision Plus Protein All Blue molecular standards (Bio-Rad) were used for determining relative migration of protein in the gel.

For Western blot analysis, proteins were transferred from SDS-PAGE gels to Immobilon-FL membranes overnight at 0.09 amps at 4°C, and the membranes were incubated with primary antibodies (1∶5,000 or 1∶10,000) for 2 h followed by 1 h incubation with the appropriate IRDye secondary antibody (1∶10,000). All fluorescence signals were detected and quantified using the Odyssey Infrared Imaging System (LI-COR Biosciences).

### N-terminal Sequencing by Edman Degradation

SDS-polyacrylamide gels were cast 24 h in advance. After electrophoresis, the separated protein bands were transferred onto Immobilon-FL overnight at 0.09 A at 4°C. The proteins were stained with CBB and excised for sequencing according to the method of Le Gendre and Matsudaira [39]. Sequence analysis was performed by Dr. Richard Cook in the Proteomics Core Laboratory at Baylor College of Medicine (Houston, TX).

All amino acid sequences refer to the rabbit RyR1 sequence (O. *cuniculus*), Swiss-Prot accession number P11716.

### Sequence Analysis

The RyR1 amino acid sequence was searched for potential nucleotide binding sites using the reported consensus nucleotide binding motifs: GXGXXG, [R/K]XXXGXXXL and Y[G/A/S/T][V/G][K/T/Q/S/N] [31], [32] with ScanProsite (http://prosite.expasy.org/scanprosite/) and EBI PRATT server (http://www.ebi.ac.uk/Tools/pratt/). Secondary structure prediction was performed using the PSIPRED Protein Structure Prediction Server (http://bioinf.cs.ucl.ac.uk/psipred/). Multiple sequence alignments were carried out using ClustalW2 (http://www.ebi.ac.uk/Tools/msa/clustalw2/) [40].

### Ethics Statement

This study was carried out in strict accordance with the recommendations in the Guide for Care of Laboratory Animals of the National Institute of Health. The protocol was approved by the Animal Welfare Committee of the University of Texas Health Science Center at Houston (Animal Welfare Assurance Number: A3413-01; protocol number: HSC-AWC-10-173). New Zealand Rabbit was used as a source of fast twitch skeletal muscle for the preparation of microsomal SR membranes and RyR1 protein. Tissue dissection was performed under anesthesia by intramuscular injection of a combination of ketamine and xylazine, and all efforts were made to minimize discomfort and suffering of the animals.

## Results

### Specific Photoaffinity Labeling of RyR1 with BioATP-HDZ

**Figure 1:**
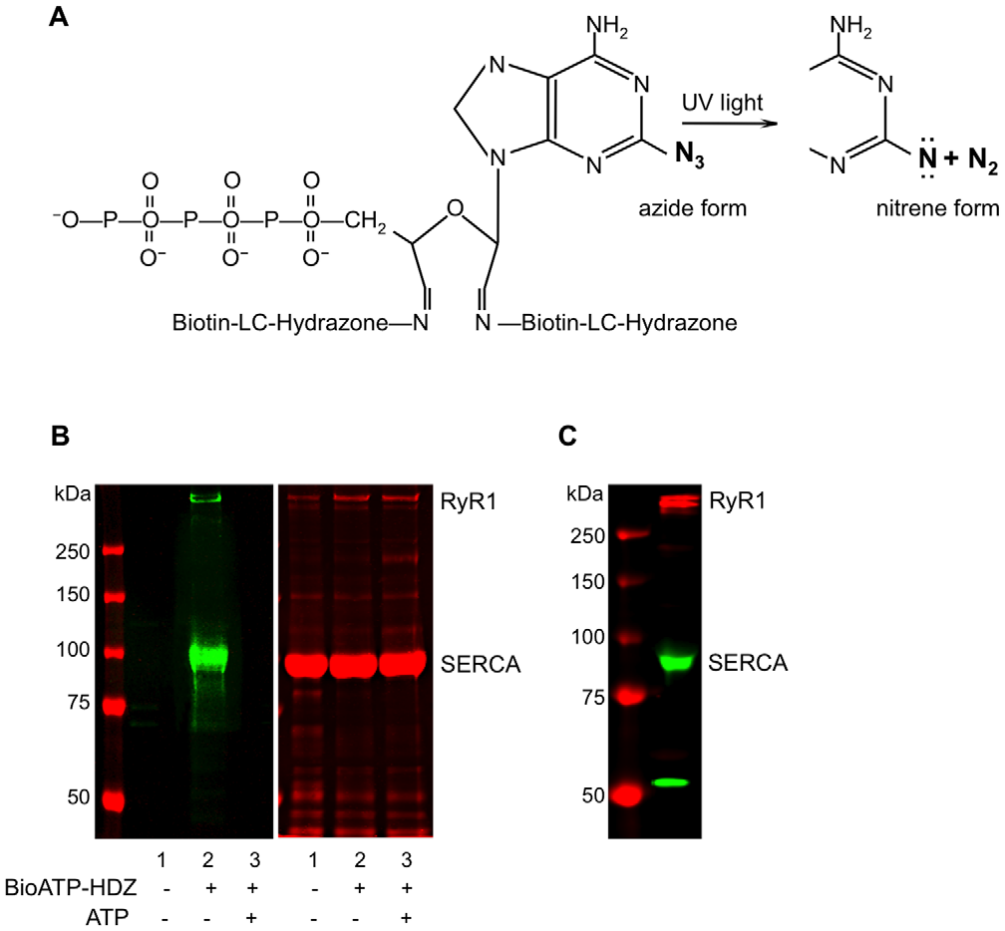
Crosslinking with BioATP-HDZ. (**A**) Structure of 2-azidoadenosine 5′-trisphosphate 2′,3′-biotin-long-chain-hydrazone (BioATP-HDZ). A highly reactive nitrene is produced by UV exposure and can form a covalent bond with a neighboring peptide backbone or amino acid side chain of the protein. (**B**) Fast-twitch skeletal muscle SR membranes were specifically labeled by 10 µM BioATP-HDZ and detected by IRDye800CW-streptavidin in-gel overlay. The left panel is the streptavidin in-gel overlay and the right panel is the CBB stain of the same gel. *Lane* *1* – non-labeled SR membranes; *lane* *2* – SR membranes labeled with BioATP-HDZ; *lane* *3* – competition of BioATP-HDZ labeling by ATP in SR membranes. (**C**) Western blot with anti-RyR1 (pseudo-colored red) and anti-SERCA2 (pseudo-colored green) antibodies.

The bifunctional ATP analog, 2-azidoadenosine 5′-trisphosphate 2′,3′-biotin-long-chain-hydrazone (BioATP-HDZ), contains an azido group that can covalently label ATP-binding sites in proteins (Figure 1A). BioATP-HDZ also contains two non-cleavable biotin moieties that are used to detect labeled polypeptides through an interaction with streptavidin. To determine if BioATP-HDZ can bind specifically to RyR1, SR membranes were incubated with 10 µM BioATP-HDZ and UV crosslinked in the absence or presence of excess ATP. The gels showed an incorporation of the biotin tag into several SR membrane proteins, including two major proteins at ∼500 kDa and ∼100 kDa (Figure 1B). We confirmed the identity of both BioATP-HDZ-labeled proteins by Western blot with sequence specific antibodies (Figure 1C): the high molecular weight band at ∼500 kDa corresponds to RyR1, and the ∼100 kDa band is sarco/endoplasmic reticulum Ca^2+^-ATPase (SERCA), one of the most abundant proteins in SR membranes. Labeling of both RyR1 and SERCA with BioATP-HDZ was specifically competed by pre-incubating the SR membranes with 200 µM of ATP (Figure 1B, lane 3). Moreover, SERCA, a well-known ATP-binding protein, provides an internal positive control for BioATP-HDZ labeling reaction.

**Figure 2:**
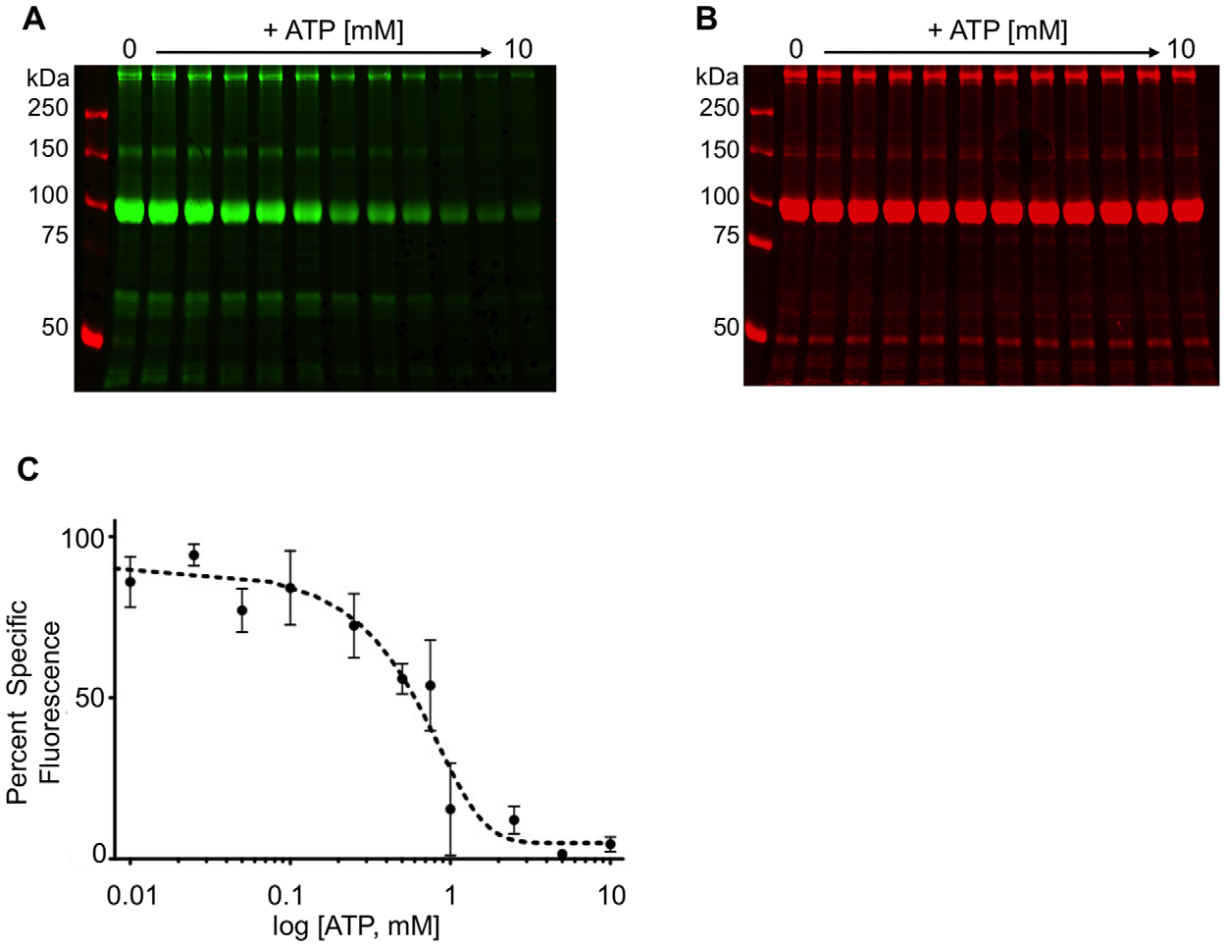
Quantification of the binding affinity of BioATP-HDZ to RyR1. SR membranes were labeled with 10 µM BioATP-HDZ in the absence or presence of increasing concentrations of ATP. The crosslinking of BioATP-HDZ to RyR1 was determined by IRDye800CW-streptavidin in-gel overlay (pseudo-colored green, **A**), and the signal was normalized to its respective CBB stain intensity at 700 nm (pseudo-colored red, **B**). (**C**) Quantification of BioATP-HDZ crosslinking to RyR1 as the mean of 3 independent experiments ± SEM. The IC_50_ determined by non-linear regression was 0.6±0.2 mM.

To determine the binding affinity of BioATP-HDZ to RyR1, competition of its binding and covalent labeling of the receptor protein was performed. In this set of experiments, SR membranes or purified RyR1 were pre-incubated with increasing amounts of ATP, followed by an incubation with 10 mM BioATP-HDZ. The binding of BioATP-HDZ to RyR1 in SR membranes was competed by ATP in a concentration-dependent manner with an IC_50_ = 0.6±0.2 mM (Figure 2). An IC_50_ = 0.95±0.1 mM was determined for purified RyR1 (Figure S1). These values are in agreement with reported EC_50_ values for activation of RyR1 with ATP (∼0.5 mM) [15], [16], [17], [18], [19], [20], [21].

### Purification of BioATP-HDZ-labeled RyR1 Tryptic Complexes

**Figure 3:**
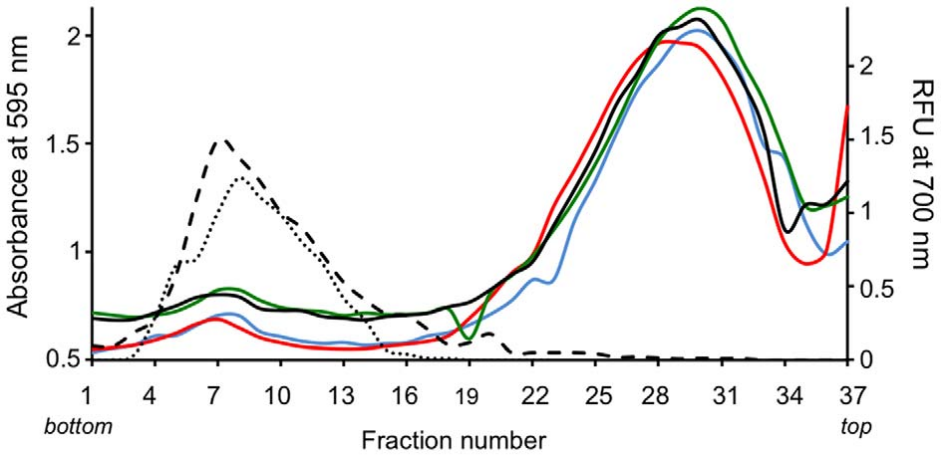
Distribution profiles of SR membrane proteins across sucrose gradients. Amount of protein estimated by absorbance at 595 nm using BioRad protein assay reagent is plotted for each fraction collected from the sucrose gradients. Experimental conditions: blue line – digested SR membranes, red line – digested SR membranes in the presence of ATP, green line – digested SR membranes crosslinked with BioATP-HDZ, black line – digested SR membranes crosslinked with BioATP-HDZ in the presence of ATP. Western blot analyses of sucrose gradient fractions with anti-RyR1 antibody detected at 700 nm (right axis): dotted line – undigested SR membranes, dashed line – digested SR membranes.

In order to purify the RyR1 fragments covalently labeled with BioATP-HDZ, the labeled full-length protein was subjected to a limited proteolysis and then semi-purified in a linear sucrose gradient. To analyze whether the covalent binding of BioATP-HDZ to RyR1 has an impact on its sedimentation profile, both BioATP-HDZ-labeled and unlabeled SR membranes were treated by trypsin under the same conditions and separated by centrifugation in density sucrose gradients. Figure 3 shows the protein distribution profile through the sucrose gradients following proteolysis under different conditions. Under our digestion conditions, the labeled RyR1 largely retained its tetrameric assembly and sedimented in the same manner as undigested RyR1. Under all experimental conditions, sucrose gradient fractions 6 through 9 contained the greatest amount of RyR1. It is notable that neither BioATP-HDZ nor ATP altered the sucrose sedimentation profile for digested RyR1.

### Identification of BioATP-HDZ-labeled Tryptic Fragments

**Figure 4:**
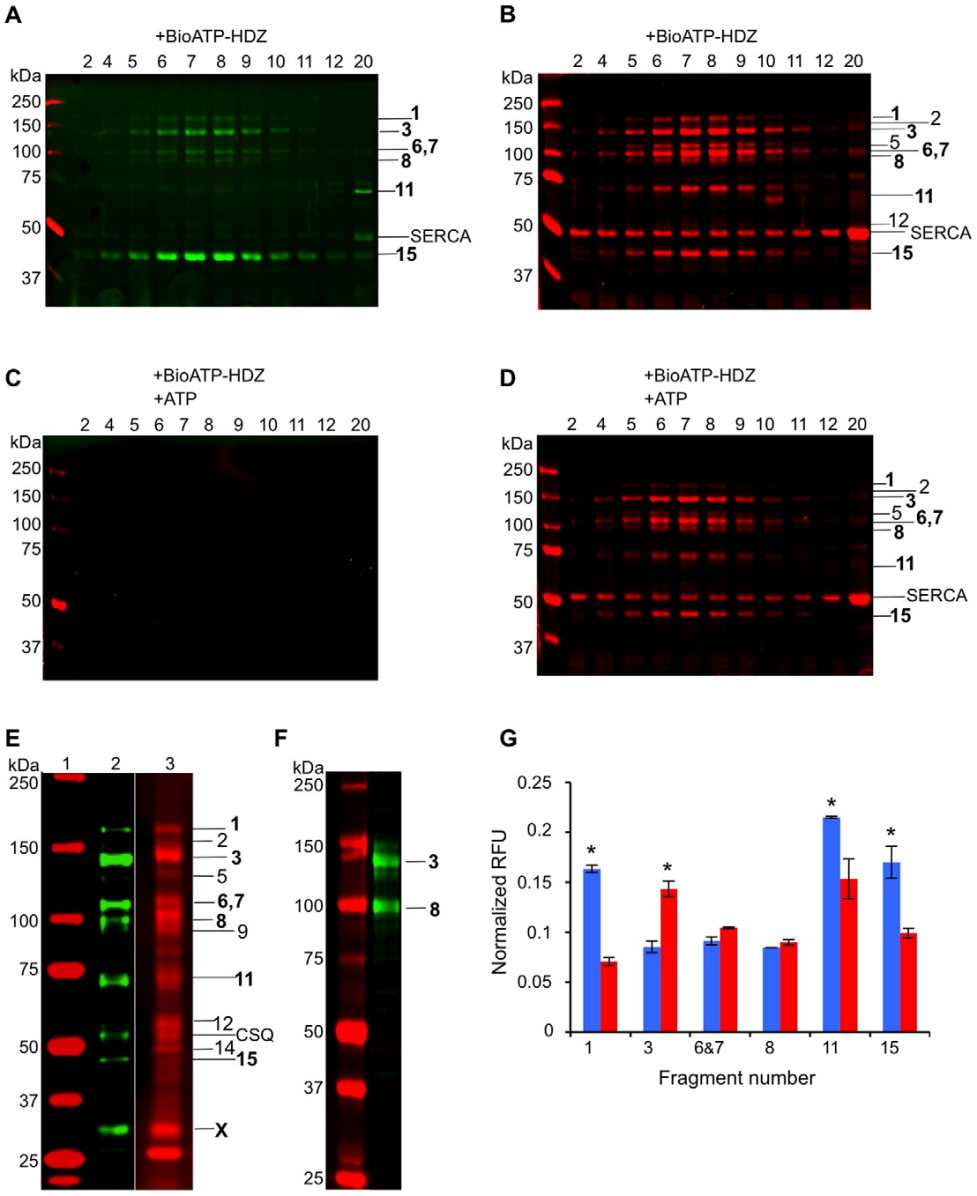
Detection of BioATP-HDZ-labeled tryptic fragments. SR membranes were labeled with BioATP-HDZ in the absence (**A**, **B**) and presence (**C**, **D**) of ATP. Following the crosslinking, the SR membranes were digested with trypsin, solubilized with 2% CHAPS and separated by sucrose gradient centrifugation. Sucrose gradient fractions were analyzed by SDS-PAGE and labeled fragments were detected by in-gel IRDye800CW-streptavidin overlay at 800 nm. (**A**, **C**). The gels were then stained with CBB and scanned at 700 nm (**B**, **D**). Sucrose gradient RyR1 peak fractions are shown; fraction 20 was analyzed as an internal control for BioATP-HDZ labeling of SERCA. The number of the sucrose gradient fraction run in each lane is labeled above gels in A–D. (**E**) Purified RyR1 crosslinked with BioATP-HDZ, digested with trypsin and transferred to immobilon-FL membrane: *lane* *1* – molecular weight standards; *lane* *2* – IRDye800CW-streptavidin overlay; *lane* *3* – CBB staining. (**F**) Immunoblotting of the trypsin-digested labeled RyR1 with a specific antibody against RyR1 amino acid sequence 416–434. (**G**) Plot of the normalized relative fluorescence (RFU) calculated for individual BioATP-HDZ-labeled tryptic fragments of RyR1 detected in SR membranes (blue) and purified RyR1 (red), error bars represent SEM (N = 3) and * indicates *p*<0.03. The amount of BioATP-HDZ specifically bound with each labeled band in (**A**) is quantified using the equation [(*F*_800_/*F*_700_)_noATP_ – (*F*_800_/*F*_700_)_ATP_], where [(*F*_800_/*F*_700_)_noATP_ is the total bound BioATP-HDZ measured as the fluorescence signal at 800 nm normalized to its respective CBB signal at 700 nm in (**B**). (*F*_800_/*F*_700_)_ATP_ estimates non-specific binding of BioATP-HDZ in (**C**) and (**D**). Calculations for SR membrane were performed using sucrose gradient fraction 7 with the exception of fragment 11, which was analyzed in fraction 20. The same quantifications were performed for the purified RyR1 shown in (**E**): *lane* *2* – total bound BioATP-HDZ, *lane* *3* – non-specific binding of BioATP-HDZ. Throughout the figure, bands detected by CBB stain and streptavidin overlay are numbered to the right of the gel by correspondence to [37], bold numbers indicate BioATP-HDZ containing bands.

**Figure 5:**
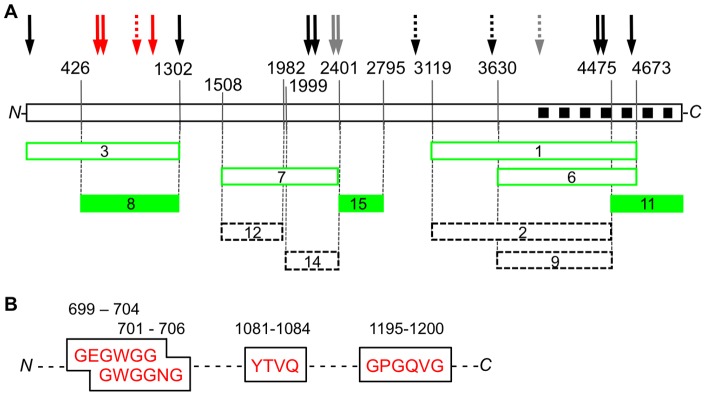
Map of BioATP-HDZ-labeled tryptic fragments of RyR1. (**A**) A linear schematic of RyR1 sequence; the black squares indicate the predicted transmembrane domains located at amino acids 3985–4937; major tryptic cleavage sites are indicated with vertical lines and corresponding amino acids are shown above. Arrows indicate locations of predicted conserved ATP-binding motifs in the primary structure of RyR1: solid arrows – Walker-A and the partial Walker-B motifs (amino acids 2–7, 699–704, 701–706, 1195–1200, 1302–1310, 2126–2234, 2264–2269, 2369–2377, 2370–2375, 4449–4452, 4452–4457 and 4602–4607), dashed arrows (amino acids 1081–1084, 2935–2938, 3503–3506 and 3937–3940) – the conserved RyR1 sequences similar to the ATP-binding site in GroES, grey arrows (amino acids 2369–2377, 2370–2375 and 3937–3940) – the motifs that were ruled out as ATP-binding sites in [34], red arrows (amino acids 699–704, 701–706, 1081–1084 and 1195–1200) – motifs that can constitute ATP-binding sites in RyR1. Observed RyR1 tryptic fragments are depicted as rectangles: dashed lined – unlabeled fragments observed only in CBB-stained gels, green – BioATP-HDZ-labeled fragments, green-filled (amino acids 427–1302, 2402–2795 and 4476–5037) – labeled fragments that can potentially constitute ATP-binding sites. (**B**) Four predicted ATP-binding motifs found in fragment 8 (amino acids 427–1302).

The peak RyR1 sucrose gradient fractions were analyzed by SDS-PAGE, and BioATP-HDZ-labeled proteolytic fragments were identified either by sequence analysis or Western blot with specific antibodies against the RyR1 sequences. Eight tryptic fragments with the apparent molecular weight of 170, 145, 110, 102, 95, 70, 50 and 45 kDa were resolved by SDS-PAGE and detected by in-gel overlay with streptavidin as polypeptides containing covalently bound BioATP-HDZ (Figure 4A and Figure S2A). The binding of BioATP-HDZ to these fragments was competed by ATP (Figure 4C). Six labeled polypeptides were identified by *N*-terminal sequencing (Table 1) as tryptic fragments of RyR1 and were consistent with the tryptic fragments 1 (170 kDa), 6 (110 kDa), 7 (102 kDa), 8 (95 kDa), 11 (70 kDa) and 15 (45 kDa) identified in [37]. To position the labeled tryptic fragments within the RyR1 amino acid sequence, their *C*-terminal ends were assigned based on the correspondence of their apparent molecular mass to the mass calculated from predicted tryptic cleavage sites in the RyR1 sequence (Table 1 and Figure 5A). Due to *N*-terminal blockage, the 145-kDa tryptic fragment was identified by Western blot with sequence-specific antibodies derived against the amino acids 416–434 of RyR1 (Figure 4F). This polypeptide was identified as fragment 3 in Zhang *et* *al.* [37] and likely contains amino acids 1–1302. The same *N*-terminal antibody also recognized the 95-kDa fragment 8 (Figure 4F) indicating that the 145-kDa fragment 3 is the precursor of fragment 8 (amino acids 427–1302).

**Table 1:** Labeled fragments of RyR1 proteolytic complex.

MW^a^ (kDa)	Proteolytic Fragments	*N*-terminal Sequence	Corresponding Sequence	MW^b^ (kDa)
170	1	TQVKGVGQN	3120–4673	176
145	3	ND^c^	1–1302	148
110	6	AVVAXFRMTP	3631–4673	118
102	7	ISHTDLVIG	1509–2399	102
95	8	GSGPPAGPAL	427–1302	99
70	11	KLGVDGEEEE	4476–5037	64
50	SERCA	AAVGNKMFVK^d^	506–923	48
53	CSQ	(E)GLDFPEYD(G)^e^	30–395	41
45	15	RREHFGEEPP	2402–2795	45
30	X	I(G)(F)(F)(L)(V)-(I)(E)(Y)^f^	-	-

a – an apparent molecular weight as determined by mobility in SDS-PAGE.

b – molecular weight of tryptic fragment calculated based on predicted trypsin cleavage sites in RyR1 sequence (Swiss-Prot accession number P11716).

c – sequence not determined (ND) due to *N*-terminal blockage.

d – SERCA sequence (Swiss-Prot accession number P04191).

e – Calsequestrin 1 sequence (Swiss-Prot accession number P07221).

f – non-identified sequence.

Interestingly, the 70-kDa polypeptide was unexpectedly discovered in an upper fraction of the sucrose density gradient and was highly labeled with BioATP-HDZ (Figure 4A and Figure S2A). This band was identified by *N*-terminal sequencing as a RyR1 proteolytic fragment comprising amino acids 4476–5037, corresponding to fragment 11 in [37]. This fragment includes several predicted membrane-spanning domains and the *C*-terminus of RyR1 [4], [5], [6]. Labeling of the 70-kDa fragment is also consistent with a previous report of [α^32^P]Bz_2_ATP crosslinking to a 76-kDa RyR1 tryptic fragment [41]. The unexpected sedimentation of the majority of fragment 11 separately from the peak fractions of the tetrameric RyR1 may likely be explained by a reduced stability of the proteolyzed channel after detergent solubilization weakening the interaction of this particular region with the rest of the protein mass. Finally, the 50-kDa tryptic fragment was sequenced and identified as the proteolytic product of SERCA and contained the ATP-binding site at amino acids 506–515 (Table 1 and Figure S3).

We found that the tryptic cleavage profile of purified RyR1 was similar to that obtained for the SR membranes (Figure 4E and Figure S2B). However, the relative amounts of BioATP-HDZ bound to the tryptic fragments were slightly different (Figure 4G). The highest efficiency of BioATP-HDZ labeling was detected for fragments 1, 11 and 15 when RyR1 was labeled within SR membranes. In experiments with the purified receptor, fragments 11 and 15 remain highly labeled, however, fragment 3 shows an increased BioATP-HDZ labeling, while a decreased labeling was observed in fragment 1. This occurrence can be due either to altered accessibility of ATP-binding sites in the solubilized channel protein or prolonged trypsin digestion of the purified RyR1.

Moreover, the digestion of BioATP-HDZ-labeled purified RyR1 resulted in the appearance of two additional tryptic fragments of 53 and 30 kDa (Figure 4E). The 53-kDa polypeptide was identified by *N*-terminal sequencing as the Ca^2+^ binding protein, calsequestrin (CSQ) (Table 1). CSQ is known to physically associate with RyR1 through interactions with triadin or junctin and co-purifies with RyR1 [42], [43]. While CSQ does not contain known ATP binding motifs, it has been found to interact with a 30 kDa ADP/ATP translocase, which is proposed to form a macromolecular complex with junctin to regulate RyR1 activity [44]. As such, the interaction of CSQ with the ADP/ATP translocase is a plausible mechanism for its labeling by BioATP-HDZ. Furthermore, CSQ was likely present in the RyR1 tryptic complexes purified from SR membranes, but its detection was occluded by the presence of an overwhelming signal from BioATP-HDZ-labeled proteolytic fragment of SERCA migrating at ∼50 kDa (Figure 4A). Since trypsinization of CSQ has been shown to be blocked by the presence of PMSF this likely explains the presence of undigested CSQ in RyR1 tryptic complexes in our experiments [45]. Sequence analysis of the 30-kDa fragment (proteolytic fragment X) (Table 1, Figure 4E) resulted in multiple amino acid sequences that did not allow for the identification of the polypeptide.

After the initial sequences of the labeled fragments were identified, each was analyzed for the potential to form an ATP-binding site. Starting from the *N*-terminus, the tryptic fragment 3 (amino acids 1–1302) contains four ATP-binding consensus motifs at amino acids 2–7, 699–704, 701–706 and 1195–1200 conforming to the Walker-A and one GroES ATP-binding consensus sequence at amino acids 1081–1084 (Figure 5A). This fragment is the precursor to the smaller fragment 8 (amino acids 427–1302), whereupon the first 426 amino acids are cleaved (Table 1 and Figure 5A). However, the expected 47-kDa fragment for the amino acids 1–426 was not detected in any tryptic fragment preparations and is likely rapidly proteolyzed into smaller polypeptides. The *N*-terminus contains one predicted ATP-binding consensus motif at amino acids 2–7 (Figure 5A). While a crystal structure of the *N*-terminal domain of RyR1 exists, these residues were not resolved leaving an open question on the structural arrangement of this region and its involvement in ATP-binding in RyR1 channel [46].

Fragment 7 (amino acids 1509–2399), the next labeled polypeptide in the RyR1 sequence, contains one conserved Walker-A motif at the amino acid sequence 2264–2269 and three partial Walker-B motifs at the amino acid sequences 2126–2134, 2369–2377 and 2370–2375 (Table 1 and Figure 5). However, earlier alanine-scanning mutagenesis studies eliminate the motifs at amino acids 2369–2377 and 2370–2375 as the ATP-binding sites responsible for activation of RyR1 [34]. In addition, we found that further proteolysis of fragment 7 generated the smaller fragments 12 and 14. While both fragments are detected in the CBB stained gel and fragment 14 retains the conserved ATP-binding consensus motifs, neither are labeled by BioATP-HDZ (Figure 4E). Given this occurrence, it is possible that fragments 12 and 14 loose their label upon tryptic digestion. Since the assignment of the *C*-terminus for each fragment was based on the correlation of observed molecular weight to a molecular weight calculated from amino acid sequence, the presence of multiple tryptic cleavage sites within the last ∼20 residues of fragments 12 and 14 leaves some ambiguity with respect to the exact position of the *C*-terminus. Thus, we cannot rule out the possibility that if the BioATP-HDZ label was at the *C*-terminus, it was lost due to further digestion of these fragments leaving an open question on the direct involvement of corresponding sequences in the formation of the ATP-binding pocket in RyR1.

Proceeding toward the *C*-terminal region, three fragments were labeled by BioATP-HDZ: fragments 1 (amino acids 3120–4673), 6 (amino acids 3631–4673) and 11 (amino acids 4476–5037) (Figure 5A). Fragment 1 contains five predicted ATP-binding consensus motifs at amino acids 3503–3506, 3937–3940, 4449–4454, 4452–4457 and 4602–4607. However, the amino acid sequence 3937–3940 was eliminated as an ATP-binding site by prior mutagenesis studies [34]. Fragment 6, the smaller proteolytic product of fragment 1, does not contain the predicted ATP-binding motif at amino acids 3503–3506, yet still remains labeled by BioATP-HDZ. Moreover, fragments 2 and 9 (amino acids 3120–4475 and 3631–4475, respectively) originating from the same RyR1 region as fragments 1 and 6, were not observed to have any BioATP-HDZ label, but were present in the CBB gels (Figure 4E). Therefore, it is reasonable to eliminate the sequences encompassing amino acids 3120–4475 as contributing to the ATP-binding pocket. Fragments 1 and 6 share an overlapping sequence with the labeled fragment 11 at amino acids 4476–4673. This region contains a putative nucleotide-binding consensus sequence at amino acids 4602–4607. However, based on topology of the transmembrane domains in RyR1 [6], this motif cannot be involved in the cytosolic regulation of RyR1 by ATP due to its location in the SR lumen. Therefore, the remaining cytoplasmic sequences within fragment 11 from amino acids 4674–5037 likely contain sequences important for binding ATP.

## Discussion

**Figure 6:**
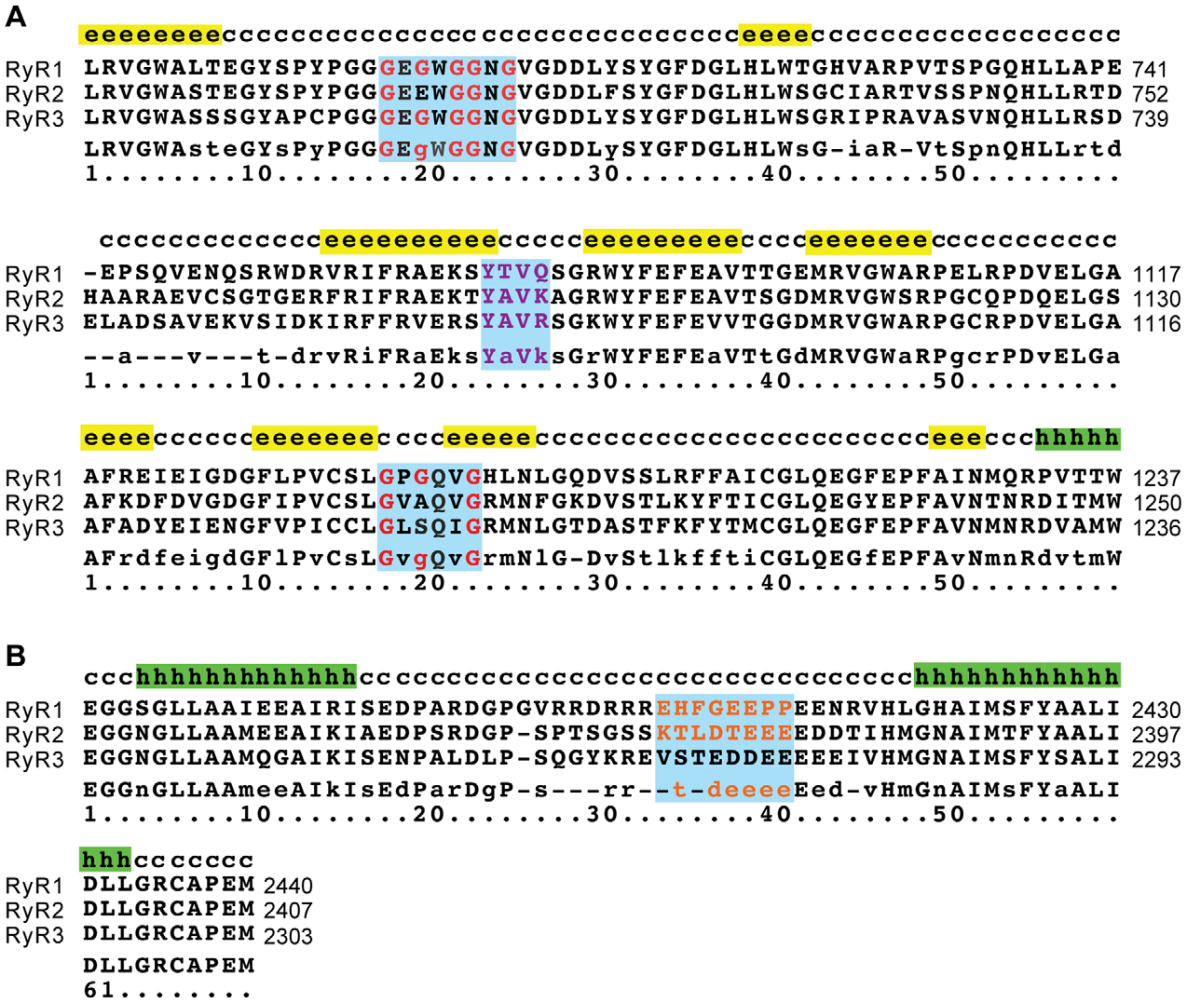
Sequence alignment of the putative ATP-binding regions in the RyR channel family. The aligned sequences are: rabbit RyR1 (GI: 134134), rabbit RyR2 (GI: 308153559), human RyR3 (GI: 126032338). Predicted secondary structure elements are given above the sequences and annotated as following: ‘e’ – β-strand, ‘h’ – α-helix; ‘c’ – coil; consensus sequences are shown below the aligned sequences with following symbols: upper-case – identical residues, lower-case – similar residues, dash – different residues. The putative ATP-binding sequences are highlighted with blue. (**A**) The ATP-binding motifs identified in the tryptic fragment 8: red – conserved glycine; purple – amino acid residues conforming to the ATP-binding motif in GroES. (**B**) The *N*-terminal sequence of fragment 15 aligned with the putative ATP-binding site in RyR2 [54] are shown in orange.

Covalent labeling by a novel, bifunctional ATP crosslinker, BioATP-HDZ, demonstrated that three regions in RyR1 are involved in ATP-binding. These regions are widely separated in the primary structure of RyR1 and comprise sequences 427–1302 (fragment 8), 2402–2795 (fragment 15) and 4476–5037 (fragment 11). While sequence analysis predicts at least 16 nucleotide-binding consensus motifs in RyR1, our crosslinking results suggest that the four ATP-binding motifs identified at amino acids 699–704, 701–706, 1081–1084 and 1195–1200 (Figure 5) can constitute a putative ATP-binding pocket. Comparison of RyR1, RyR2 and RyR3 sequences revealed that locations of the *N*-terminal ATP-binding consensus motifs and their surrounding sequences are conserved (Figure 6A). In the three RyR isoforms, secondary structure prediction for these regions showed flanking β-strands connected by a loop that together can form a cleft in which ATP binds. This ATP-binding structural motif (the so-called ‘ATP-grasp fold’) has been identified in multiple proteins [47]. The IP_3_ receptor (IP_3_R) family of Ca^2+^ release channels, closely related to RyR channels, is also essentially regulated by ATP (reviewed in [48]). Two ATP-binding regions have been determined in IP_3_R1 and one region in IP_3_R3 by covalent labeling with 8-azido-[α-^32^P]ATP. These regions were found to contain glycine-rich conserved motifs [49]. However, the sequences comprising the ATP-binding sites in IP_3_Rs do not align with any of the putative ATP-binding sequences identified in RyRs. These differences likely underlie the distinctive modulation of RyR and IP_3_R channels by nucleotides.

While four ATP-binding motifs were found within fragment 8, fragment 15 did not contain any known nucleotide-binding consensus motifs, however it showed a high capability for crosslinking with BioATP-HDZ. We propose that the labeling of fragment 15 occurred due to its contribution to the 3D architectural arrangement of an ATP-binding pocket or, alternatively, through the presence of a non-canonical ATP-binding site. There are numerous examples where sequences that do not include the canonical Walker-A or Walker-B motifs alone are still important for high-affinity ATP-binding [50], [51], [52], [53]. Moreover, the *N*-terminal sequence of fragment 15 aligns well with the amino acid sequence 2371–2378 in RyR2 (Figure 6B) that was observed to bind ATP with a *K_d_* ∼0.03 mM [54]. The same consideration applies to fragment 11, where the predicted ATP-binding consensus motif (amino acids 4602–4607) can be ruled out based on its topology with respect to the SR membrane [6]. The labeling of fragment 11 was likely due to the close proximity of its cytoplasmic sequences to the ATP-binding pocket. One potential scenario would be for the last ∼100 residue cytoplasmic *C*-terminal ‘tail’ to architecturally contribute to the ATP-binding pocket located distantly from the channel pore. Furthermore, excessive labeling of fragments 11 and 15 compared with other proteolytic fragments (Figure 4G) might be due to the particular architectural arrangement of these sequences within an ATP-binding pocket that makes them readily accessible to the formation of covalent bond with the reactive nitrene of BioATP-HDZ.

**Figure 7:**
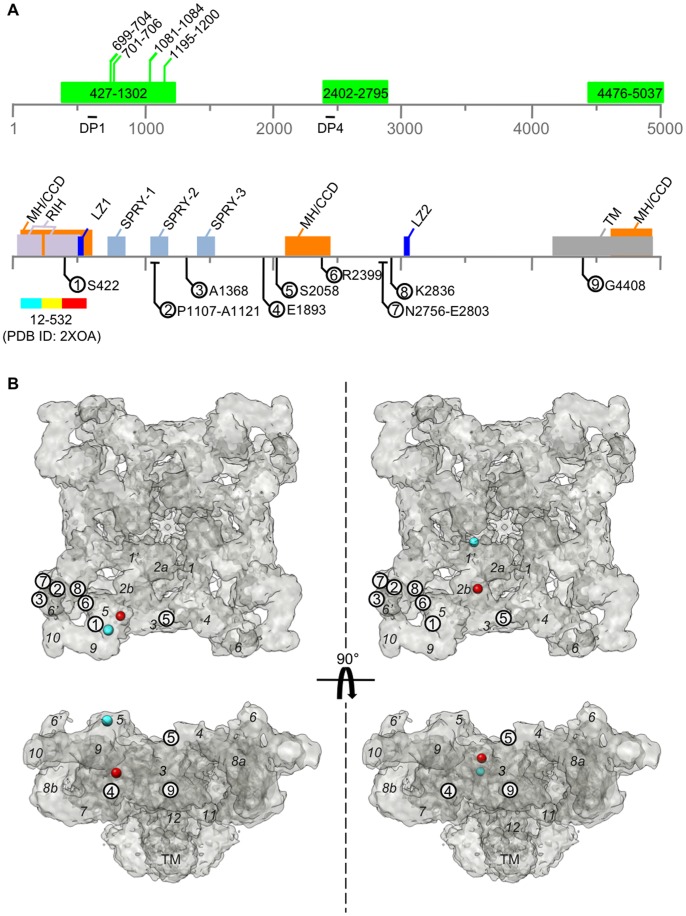
Structural arrangement of RyR1 functional sites. (**A**) A linear schematic representation of the RyR1 amino acid sequence shows the three putative ATP-binding regions (green) with the predicted ATP-binding motifs (top panel). Positions for the peptides DP1 (amino acids 590–628) and DP4 (amino acids 2442–2477) are shown [55]. Several functional domains are represented in the linear sequence of RyR1 labeled in the bottom panel: MC/CCD – mutation hot-spot for malignant hyperthermia and central core disease, RIH – RyR/IP_3_R homology regions, SPRY – SplA/RyR domain, LZ – leucine zippers, TM – transmembrane region. Sequences that were mapped in the 3D structure are indicated by the circled numbers [59], [60], [61], [62], [72]. (**B**) Surface representation of 1 nm resolution cryo-EM density map of RyR1 [63], [64] is shown for the top and side views. Left panels – computational localization of 2XOA in the clamp region, right panels – computational localization of 2XOA in the central region. The *N*- and *C*-terminus of the crystal structure of the *N*-terminal region (PDB ID: 2XOA) are represented as cyan and red spheres. The italic numbers indicate segmented subregions for one putative subunit. Subregions 1 and 6 are points where the segmentation of individual RyR1 subunits is ambiguous [64], suggesting two alternative separations where either subregions 1 and 6, or 1′ and 6′ are included within the same subunit along with the remaining subregions. Circled numbers represent the location of the corresponding sequence in (**A**) mapped in the 3D structure. For technical reasons, localizations via GFP insertions were performed in RyR2, however due to the high sequence conservation between the two isoforms and near identical 3D structures [73], [74] the results are directly transferrable to RyR1 and the analogous RyR1 sequences are shown.

Therefore, our results suggest that the putative ATP-binding sequences are located within the *N*-terminal third of the RyR1 sequence that contributes to the 3D structure of the cytoplasmic region. It is well established that the cytoplasmic region of Ca^2+^ release channels forms a structural platform for binding multiple intracellular ligands, including ATP, that modulate the gating of the channel pore (reviewed in [10]). However, the precise structural mechanism allowing the passage of Ca^2+^ ions through these channels remains poorly understood largely due to our currently limited knowledge about the location, molecular composition and 3D architecture of ligand-binding sites in RyR1. The ATP-dependent RyR1 activation is demonstrated to be achieved via global conformational changes in the channel structure that are tuned by interactions among multiple domains within the cytoplasmic region to allow the propagation of the local ligand-induced signal to the channel's gate [36]. One model for channel regulation involves the interaction of two non-continuous domains. RyR1 peptides DP1 (amino acids 590–628) and DP4 (amino acids 2442–2477) were shown to activate the channel and to increase its sensitivity to agonists [55]. Although separated by ∼2000 residues, the *N*-terminal and central regions are proposed to physically interact with each other forming a domain switch necessary for stabilizing the closed state of the channel [55]. Mutations found within these regions and associated with malignant hyperthermia and central core disease, are likely to weaken the interaction between the domains, resulting in channel destabilization and Ca^2+^ leak. Our results of BioATP-HDZ crosslinking within fragments 8 (amino acids 427–1302) and 15 (amino acids 2402–2795) are consistent with this model of a direct interaction between the *N*-terminal and central regions (Figure 7).

Further, utilizing results from cryo-EM studies that pursued 3D mapping of the RyR1 sequence, we have analyzed the identified ATP-binding regions in the context of 3D architecture of the RyR1 tetramer [56], [57], [58], [59], [60], [61], [62]. The crystal structure of the *N*-terminal region of RyR1 (amino acids 12–532) has been recently localized into the central cytoplasmic domains by fitting into the cryo-EM density map of the full-length RyR1 resolved at ∼1 nm resolution [46], [63]. The alternative location within the clamp region was determined in an earlier study by fitting of the cryo-EM derived homology model [64]. While both the homology model and the crystal structure represent roughly the same RyR1 region and agree well overall, there are slight differences in their tertiary structures that cause them to dock to different locations. However, the crystal structure does not completely match the density in the central region, leaving significant ambiguities in many minds on this matter. This discrepancy cannot be unequivocally resolved until a higher resolution structure of the full-length channel is obtained and structural validation methods discriminate between these two fits. Therefore, we have considered both locations of the *N*-terminal domain in our analysis of spatial relations between the identified putative ATP-binding sites and other functional domains mapped in the 3D structure of RyR1 (Figure 7). When the *N*-terminal domains are docked to the clamp location, the *N*-terminus is situated between domains 5 and 9 (Figure 7B, left panels). This is consistent with the previous mapping of the *N*-terminus using molecular tag insertions in expressed channels (Figure 7B) [58], [65]. While considering the central localization, the *N*-terminus is positioned within domain 1′ and is consistent with an alternative subunit segmentation (Figure 7B, right panels). The *C*-terminus is localized at the interface of domains 7 and 9 when considering the clamp localization or alternatively at domain 2b in the central localization. Furthermore, the SPRY2 domain, (amino acids 1085–1208), which includes part of fragment 8, was localized to domain 6 by an antibody to the amino acid sequence 1107–1121 (Figure 7, label 2) [61]. The SPRY2 domain contains a Walker-A motif (amino acids 1195–1200) and is preceded by the GroES ATP-binding consensus motif (amino acids 1081-1084). SPRY domains have a unique β-sandwich structure of 4 anti-parallel β-sheets and are likely involved in protein-protein and inter-domain interactions, although their function in RyR1 is still unknown [66]. Therefore, the 3D cryo-EM mapping studies along with either localization of the *N*-terminal structure place the ATP-binding sites identified within fragment 8 in close proximity to the clamp region.

The location for fragment 15 (amino acids 2402–2795) can be approximated by the previously mapped residues R2399 and K2836 flanking fragment 15 and by labeling the region including amino acids 2756–2803 with a sequence specific antibody (Figure 7, labeled 6, 8 and 7) [57], [60] This suggests a putative location for fragment 15 close to the clamp region.

Fragment 11 (amino acids 4476–5037) contains several of the transmembrane helices, which are restricted to the membrane-spanning portion of the channel (Figure 7B). A GFP insertion at G4408, which is within the extended cytoplasmic loop region, was mapped to domain 3 (Figure 7B) [57]. This helps to approximate the location of the cytoplasmic portions of fragment 11 within the RyR1 structure (Figure 7, label 10). While this location is quite distant from the clamp region, it is still conceivable that fragment 11 may communicate with the two other ATP-binding regions via its extended cytoplasmic sequences.

Thus, our crosslinking results, taken in the context of the 3D mapping studies, place the putative ATP-binding pocket near the clamp region. We propose that the *N*-terminal fragment 8 together with the two other labeled regions (fragments 11 and 15) can constitute an ATP-binding pocket within the RyR1 tetramer. Considering the individual subunit boundaries in the RyR1 tetramer determined in our earlier study [64], it is conceivable that the ATP-binding pocket is formed at the interface between two neighboring subunits. The clamp regions undergo substantial conformational changes upon channel activation and have been implicated to interact with DHPR during excitation-contraction coupling of the muscle [67]. The presence of the ATP-binding site in this location may add an additional layer of control within important channel regulatory domains localized to the clamp region. Given that RyR1 has no detectable ATP hydrolytic activity, the direct binding of ATP affects the channel function by transferring its binding energy into conformational changes of the channel that involves inter-domain, as well as inter-subunit interactions.

Another potential region for localization of the ATP-binding site is a β-sheet domain identified in the column region of the cryo-EM density map of RyR1 resolved at ∼1 nm resolution [64]. The column region is the only direct link between the cytoplasmic and transmembrane domains in the RyR1 structure, which suggests its importance in transmitting of modulatory ligand-binding signals from cytoplasmic domains to the gating pore of RyR1. The structural arrangement of this region within the 3D structure of RyR1 is reminiscent of the cytoplasmic pore of Kir channels, which binds but does not catalyze ATP [68], [69], [70], [71]. Based on this structural similarity, it is possible that the column region can constitute one of the ATP-binding sites in RyR1.

The stimulatory affect of ATP is observed only in the presence of micromolar Ca^2+^, suggesting that interplay between these two ligand-binding events underlies the stabilization of the open state of the RyR1 channel. Our results demonstrate the presence of at least four ATP-binding sites in the RyR1 tetramer under low Ca^2+^ conditions. Given the relatively low affinity of RyR1 for ATP, in order to bind ATP under physiological conditions the ATP-sensitivity of RyR1 is likely to be tuned by Ca^2+^ through an unknown mechanism. To further advance a mechanistic understanding of the coupled affect of Ca^2+^ and ATP on the channel gating, future studies will be necessary to investigate ATP-binding sites in RyR1 in the presence of channel modifiers and additionally experiments utilizing mass spectrometry will be able to address a more precise mapping of the RyR1 sequences that participate in ATP binding.

In summary, our results provide a structural context for understanding ATP binding to RyR1. While we propose that the putative ATP-binding conserved motifs within fragment 8 (amino acids 427–1302) provide a structural basis for the ATP-binding pocket, two other heavily labeled fragments 15 and 11 (amino acids 2402–2795 and 4476–5037, respectively) could play a part in the 3D conformational arrangement of the same ATP-binding pocket or constitute non-canonical ATP-binding sites in RyR1. The proposed location of the nucleotide-binding pocket in RyR1 provides new insight into the structural basis of ATP modulation of RyR1 through the interaction of the ATP-binding site with regulatory domains that affects Ca^2+^ channel gating and disease mechanism.

## Supporting Information

Figure S1 **Quantification of the binding affinity of BioATP-HDZ to purified RyR1.** RyR1 was labeled with BioATP-HDZ in the absence or presence of increasing concentrations of ATP. The crosslinking of BioATP-HDZ to RyR1 was determined by in-gel IRDye800CW-streptavidin overlay (**A**) and was normalized to its respective CBB intensity at 700 nm (**B**). (**C**) Quantification of BioATP-HDZ crosslinking to RyR1. The IC_50_ determined by non-linear regression was 0.95±0.1 mM.
(TIF)

Figure S2 **Trypsin digestion profiles of BioATP-HDZ-labeled RyR1.** (**A**) 4–12% SDS-PAGE of sucrose gradient fractions 7 (lane 2) and 20 (lane 3) from trypsin-digested BioATP-HDZ labeled SR membranes. (**B**) 4–12% SDS-PAGE of labeled purified RyR1 (lane 2) digested with trypsin. Shown is the in-gel IRDye800CW-streptavidin overlay.
(TIF)

Figure S3 **X-ray structure of SERCA bound with AMP-PCP.** Shown is a ribbon diagram of SERCA (PDB ID: 3FPB), amino acid sequence 506–515 labeled by BioATP-HDZ and identified by *N*-terminal sequencing is depicted in red; bound AMP-PCP is green.
(TIF)
