# Supplementary figures and images for: Identification of ATP-Binding Regions in the RyR1 Ca2+ Release Channel
Source: PLoS One. 2012 Nov 7;7(11):e48725. doi: 10.1371/journal.pone.0048725 (PMC3492408; doi:10.1371/journal.pone.0048725)

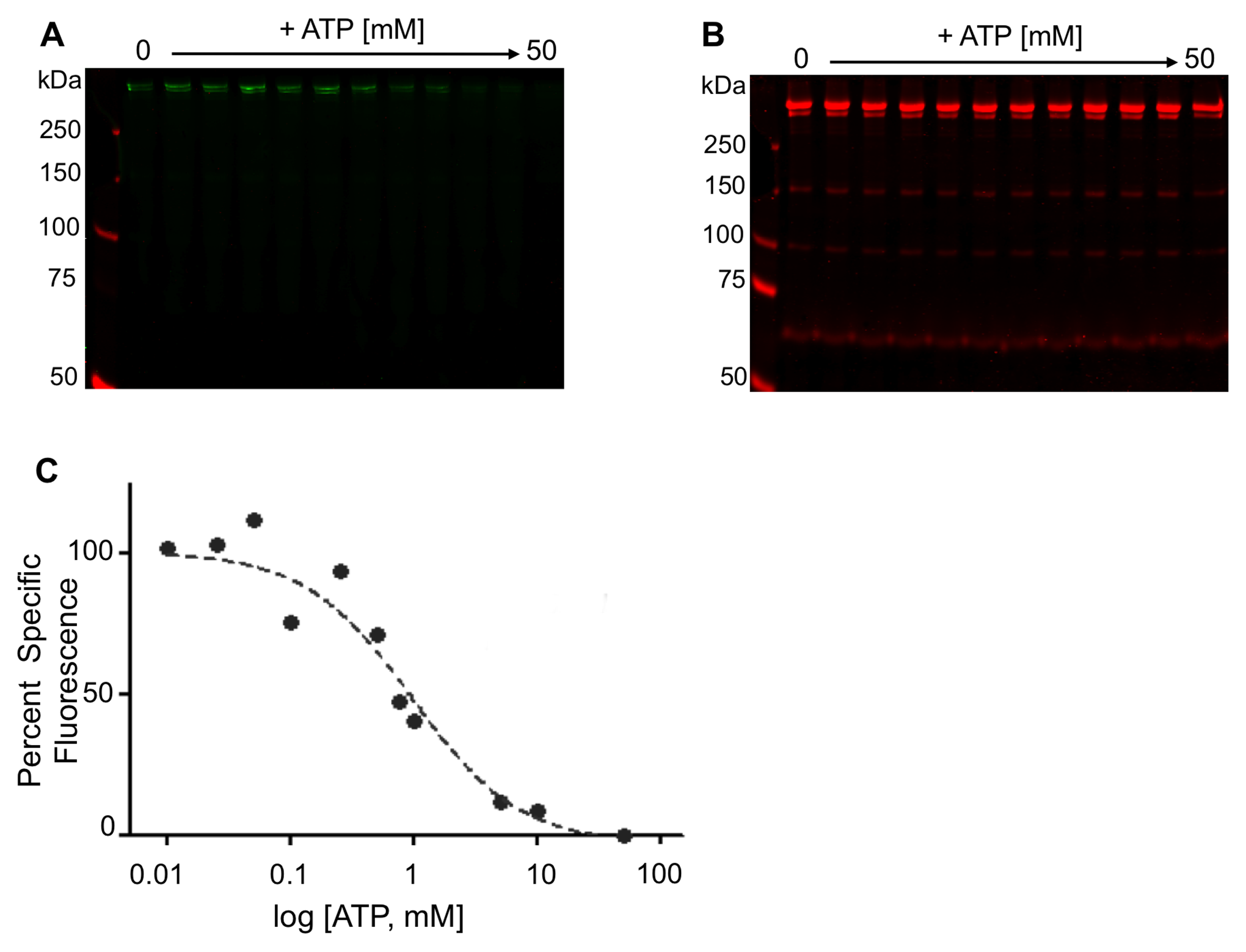

Supplement: Figure S1 — Quantification of the binding affinity of BioATP-HDZ to purified RyR1. RyR1 was labeled with BioATP-HDZ in the absence or presence of increasing concentrations of ATP. The crosslinking of BioATP-HDZ to RyR1 was determined by in-gel IRDye800CW-streptavidin overlay (A) and was normalized to its respective CBB intensity at 700 nm (B). (C) Quantification of BioATP-HDZ crosslinking to RyR1. The IC50 determined by non-linear regression was 0.95±0.1 mM. (TIF) [file pone.0048725.s001.tif]

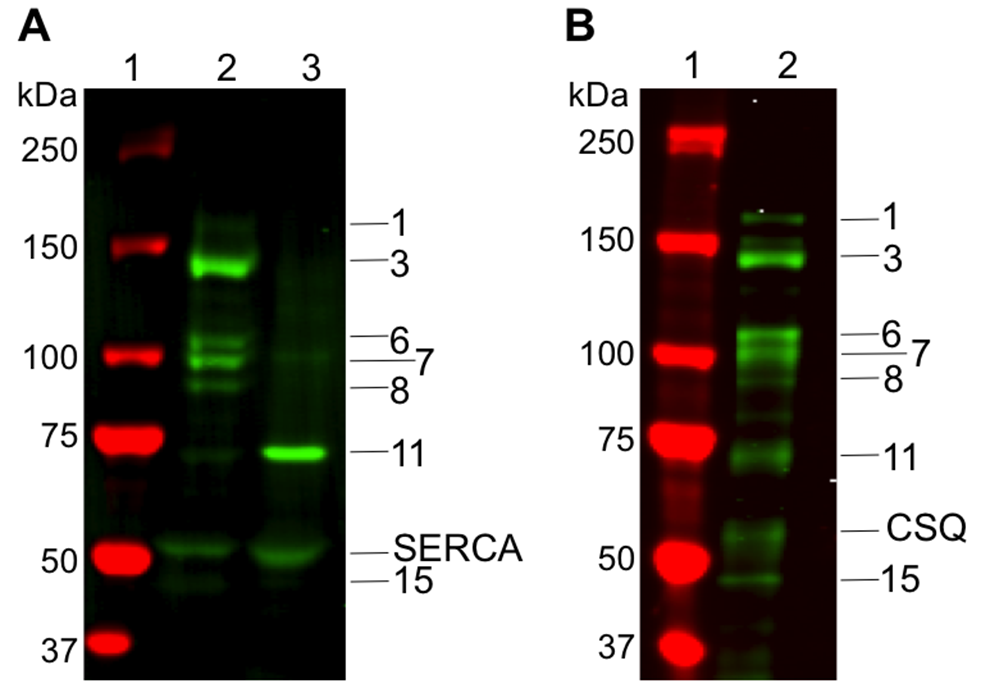

Supplement: Figure S2 — Trypsin digestion profiles of BioATP-HDZ-labeled RyR1. (A) 4–12% SDS-PAGE of sucrose gradient fractions 7 (lane 2) and 20 (lane 3) from trypsin-digested BioATP-HDZ labeled SR membranes. (B) 4–12% SDS-PAGE of labeled purified RyR1 (lane 2) digested with trypsin. Shown is the in-gel IRDye800CW-streptavidin overlay. (TIF) [file pone.0048725.s002.tif]

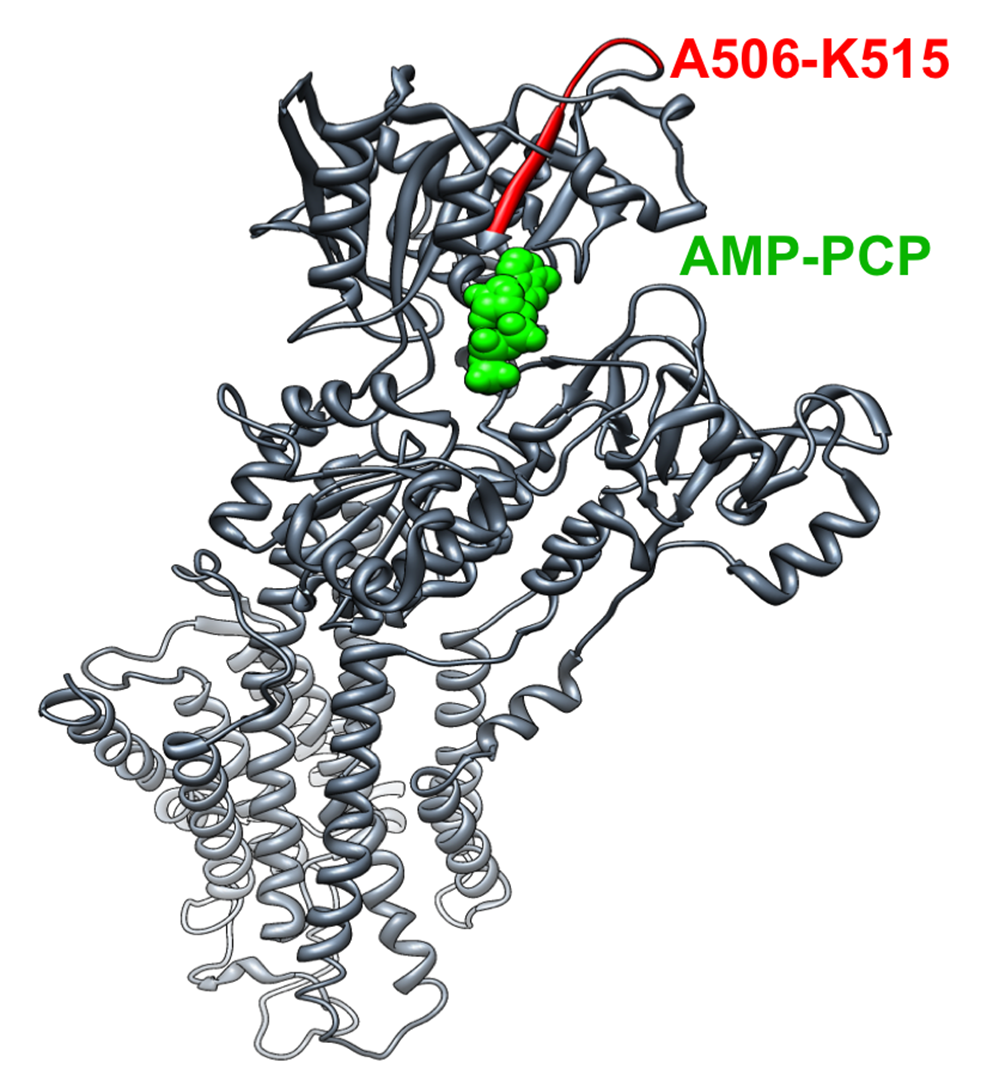

Supplement: Figure S3 — X-ray structure of SERCA bound with AMP-PCP. Shown is a ribbon diagram of SERCA (PDB ID: 3FPB), amino acid sequence 506–515 labeled by BioATP-HDZ and identified by N-terminal sequencing is depicted in red; bound AMP-PCP is green. (TIF) [file pone.0048725.s003.tif]
